# A *KIT* Variant Associated with Increased White Spotting Epistatic to *MC1R* Genotype in Horses (*Equus caballus*)

**DOI:** 10.3390/ani12151958

**Published:** 2022-08-02

**Authors:** Laura Patterson Rosa, Katie Martin, Micaela Vierra, Erica Lundquist, Gabriel Foster, Samantha A. Brooks, Christa Lafayette

**Affiliations:** 1 Etalon, Inc., Menlo Park, CA 94025, USA; khoefs@etalondx.com (K.M.); mvierra@etalondx.com (M.V.); elundquist@etalondx.com (E.L.); gfoster@etalondx.com (G.F.); 2 Department of Animal Science, UF Genetics Institute, University of Florida, Gainesville, FL 32610, USA; samantha.brooks@ufl.edu

**Keywords:** dominant white, white pattern, Arabian horse, American Quarter Horse, American Paint Horse, chestnut

## Abstract

**Simple Summary:**

Although over 40 genetic variants are known to influence white spotting and markings on the horse, many still have unknown genetic causes. Furthermore, some seem to be influenced by pigmentation (base coat color) genes. We investigated two horses demonstrating a heritable white spotting pattern of no known genetic cause. Through sequencing of the coding region of candidate genes, we identified a mutation in the *KIT proto-oncogene, receptor tyrosine kinase* (*KIT*) gene changing the coded amino acid, predicted to be deleterious to protein function. We further evaluated this variant in a population of 147 horses, characterized using photographs scored by three independent observers using a standardized Average Grade of White score. The *KIT* mutation is significantly associated with a quantitative increase in white pattern (*p* = 3.3 × 10^−12^) and demonstrates an influence of the *MC1R Extension* locus. We also report a complete link between the previously reported *KIT W19* allele and this mutation. We propose to name this mutation *W34*, following established nomenclature. Given the quantitative effect on white markings and *MC1R* influence, genetic testing for this allele can be of value for horse owners that desire to select for white patterns.

**Abstract:**

Over 40 identified genetic variants contribute to white spotting in the horse. White markings and spotting are under selection for their impact on the economic value of an equine, yet many phenotypes have an unknown genetic basis. Previous studies also demonstrate an interaction between *MC1R* and *ASIP* pigmentation loci and white spotting associated with *KIT* and *MITF*. We investigated two stallions presenting with a white spotting phenotype of unknown cause. Exon sequencing of the *KIT* and *MITF* candidate genes identified a missense variant in *KIT* (*rs1140732842*, NC_009146.3:g.79566881T>C, p.T391A) predicted by SIFT and PROVEAN as not tolerated/deleterious. Three independent observers generated an Average Grade of White (AGW) phenotype score for 147 individuals based on photographs. The *KIT* variant demonstrates a significant QTL association to AGW (*p* = 3.3 × 10^−12^). Association with the *MC1R Extension* locus demonstrated that, although not in LD, *MC1R e*/*e* (chestnut) individuals had higher AGW scores than *MC1R E*/*-* individuals (*p* = 3.09 × 10^−17^). We also report complete linkage of the previously reported *KIT W19* allele to this missense variant. We propose to term this variant *W34*, following the standardized nomenclature for white spotting variants within the equine *KIT* gene, and report its epistatic interaction with *MC1R*.

## 1. Introduction

The easily observed expression of novel mutations causing white spotting phenotypes provides straightforward targets in studies of equine genetic variation. Forty-four genetic variants, most of which are located in the *KIT proto-oncogene, receptor tyrosine kinase* (*KIT*) and the *melanocyte inducing transcription factor* (*MITF*) genes, are implicated in white spotting and depigmentation phenotypes in the horse (*W1*-*W28, W30-W33* and *SB1* on *KIT*; *SW1*, *SW3*, *SW5*, *SW6* and *SW7* on *MITF*; *SW2*, *SW4*, *LWO*, *TO* and *GR* on other genomic locations) [1,2,3,4,5,6,7,8,9,10,11,12,13,14,15,16]. Phenotypes for white spotting alleles vary from white markings on the legs and extremities, as observed with *KIT ^W20^*, to large white patches on the body, legs and head or completely white phenotypes in a few other mutant *KIT, MITF* or multiallelic individuals. Occasionally, carriers of well-documented white spotting alleles may present no to minimal white markings uncharacteristic of the spotting variant, a condition sometimes named “crypto” for its cryptic expression [17].

Loci likely altering the eumelanin vs. pheomelanin pigment proportion are also associated with the extent of depigmentation observed in the horse. The “chestnut” coat color, caused by a *Melanocortin 1 Receptor* (*MC1R*, termed the “*Extension*” locus, symbol “*E*” or “*e*”) loss-of-function mutation (*e*/*e*) and resulting in predominantly pheomelanin pigmentation, displays greater *KIT*-associated white markings [18,19,20]. Comparatively, “black” and “bay” coat colors, possessing the dominant eumelanin-producing functional *MC1R* (*E/-*) [20], demonstrate greater *MITF*-associated white markings [18]. Interactions between *KIT* and *MC1R* could be due to linkage, as both genes are located on *Equus caballus Autosome 3* (ECA3), although separated by ~42 Mbps and a centromere [21,22,23]. Alternatively, the *e*/*e* genotype may decrease melanocyte quantity or migration, intensifying the white spotting QTL effect of *KIT* mutations [18].

White markings and spotting phenotypes are varying selection in some horse breeds, depending on breeding goals and registry requirements. For the American Paint Horse, a white spotting phenotype enables registration in the “Regular” registry rather than the “Solid Paint-Bred” registry, significantly impacting the economic value of the horse [8,24]. The American Quarter Horse Association did not previously allow registration of horses with white spotting phenotypes (a rule that changed in 2004), yet statements noting white spotting as “undesirable” and “uncharacteristic” are maintained in the regulations (AQHA Official Handbook, 67th Edition, 2019). Therefore, white markings or spotting phenotypes with unknown/novel associated genetic loci are of commercial interest for the equine industry, encouraging further studies on these variants.

We describe the investigation of a white spotting phenotype (extended white markings on limbs and the ventral thoracic region), yet negative for all published white variants (*W1*-*W28, W30-W33*, *SW1*-*SW7*, *LWO*, *SB1* and *TO*) [1,2,3,4,5,6,7,8,9,10,11,12,13,14,15,16]. Using a quantitative white score phenotype, we tested the association of this allele along with the *MC1R Extension* and *ASIP Agouti* loci in 41 cases and 94 breed-matched controls lacking published white variants. We report the significant association of a missense *KIT* polymorphism with a quantitative increase in white spotting on the coat, as well as the epistatic interaction of this allele with the *MC1R* loss-of-function genotype (chestnut). We also report the complete linkage of the previously published *KIT* c.1322A>G; p.(Y441C) (*W19*) allele with this variant in 12 genotyped individuals.

## 2. Materials and Methods

### 2.1. Horses and Putative Candidate Variant Inspection

**Figure 1:**
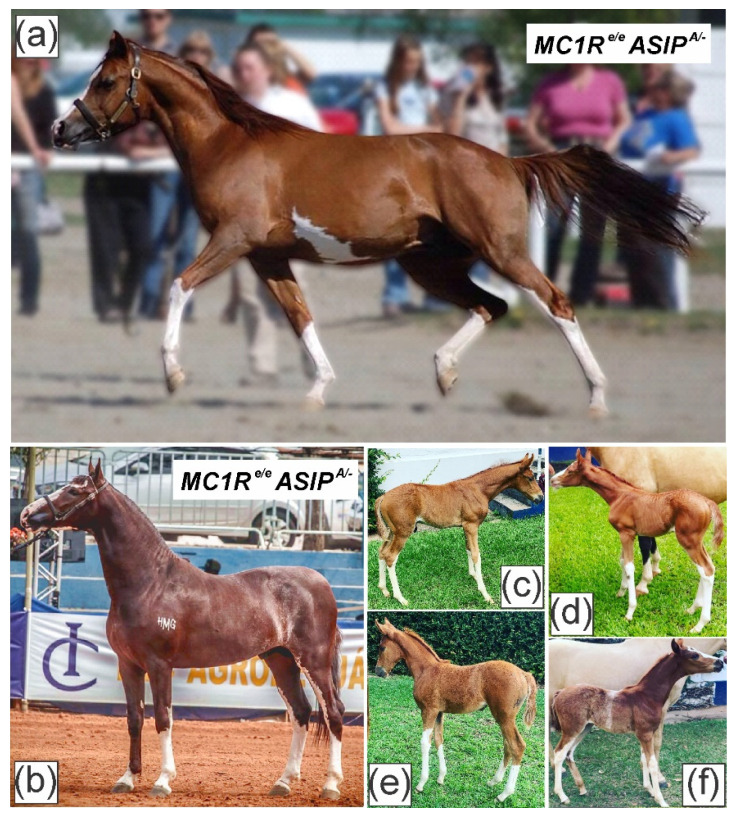
The subject stallions (**a**,**b**), demonstrating spotting phenotype extending past the distal part of the carpal and tarsal joints, as well as a facial white marking extending from the forehead to the upper and bottom lips along homozygote *MC1R e*/*e* phenotype, (**a**) also possess ventral white markings extending past the ribcage; (**c**–**f**) the respective offspring of the Mangalarga (**b**) stallion demonstrating the heritable phenotype.

An Arabian and a Mangalarga stallion, submitted to Etalon Diagnostics (Menlo Park, CA, USA) for commercial genotyping services, demonstrated a notable white spotting phenotype but no spotting or depigmentation alleles at any of the 44 known loci (*GR*, *W1*-*W33*, *SW1*-*SW7*, *LWO*, *SB1* and *TO*) [1,2,3,4,5,6,7,8,9,10,11,12,13,14,15,16]. Both individuals exhibited white markings extending past the distal part of the carpal and tarsal joints, as well as facial white markings extending from the forehead to the upper and lower lips. Additionally, the Arabian showed a large white spot on the ventral region of the body. The Mangalarga stallion was also reported to produce similar white phenotypes on its offspring (Figure 1). Given the likely heritable phenotype, we pursued further evaluation of coding regions of candidate genes *KIT* and *MITF* (known to cause phenotypically similar white spotting patterns in the horse), using previously described methods of targeted exon sequencing and alignment [13].

Putative candidate polymorphisms were further evaluated by predicting functional impacts using the PROVEAN [25] and SIFT [26] webtools, using the NCBI Equus Caballus annotation release 103. To test for associations between novel variants and white spotting phenotypes, while controlling for confounding effects originating from other known white pattern variants, 41 unrelated individuals carrying only the candidate variant, as well as 94 negative control individuals that did not possess other known white spotting or depigmentation alleles (of 1431 previously genotyped by Etalon Diagnostics [27] representing the general horse population) having submitted photographs, were selected for further phenotyping (*n* = 135). Following genotypic selection for the novel candidate variants, we observed that all *W19* individuals (*n* = 12, four times the number of individuals in the original *W19* publication) in the Etalon Diagnostics genotyped population possessed at least one allele of the candidate *KIT* variant; thus, we performed a second analysis including this allele (*n* = 147).

### 2.2. Linkage Disequilibrium Analysis with Dominant White 19 and MC1R

Due to the co-localization of loci on the *Equus caballus* autosome 3 (ECA3), we calculated the linkage disequilibrium (LD) between candidate *KIT* loci, *W19* and the *MC1R* polymorphism using Haploview V4.1 (Broad Institute, MIT and Harvard), in the 147 phenotyped individuals.

### 2.3. Phenotyping and Statistical Analysis

**Figure 2:**
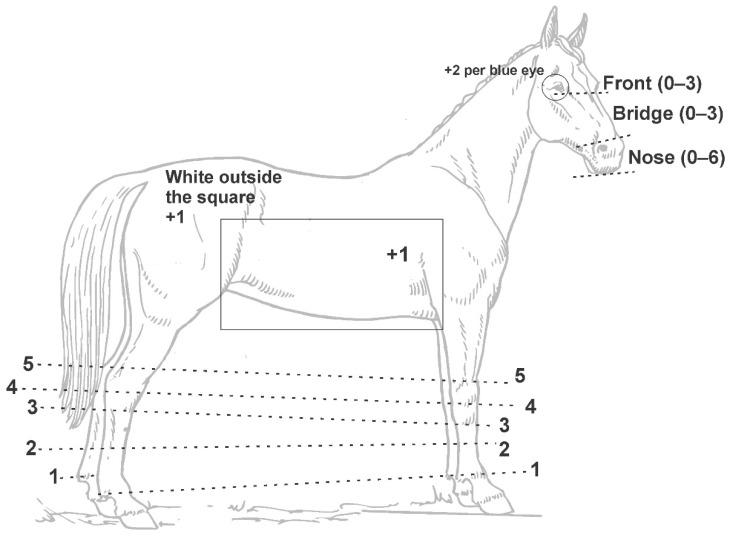
Average Grade of White phenotyping system as modified from that published by Rieder et al. [19]. Dashed lines demonstrate anatomical locations and limits for each score. Leg scores range from 0 (no white) to 5 (white above the respective dotted line).

Three blinded observers (LPR, KM and EM) scored the white phenotypes based on anonymized photographs of individual horses, following a previously published procedure for white pattern scoring [19]. In short, photos submitted by the owners were reviewed by KM and EM, selecting for 1 or 2 photographs that best represented the individual, where all 4 legs, the front of the head and any ventral/lateral white were visible for scoring purposes. Aside from the 12 compound *KIT W19* individuals, only two horses (EdX1476 and EdX4739) showed uneven body markings; these were scored based on the side with the highest amount of white. We then scored the amounts of white on the head, legs and body for each individual horse, which were then combined in a total score for each observer, summed, then averaged by the number of observers (*n* = 3), generating the “Average Grade of White” (AGW) value [19]. We modified the original score, awarding one point for white within a square comprising the ventral thorax and ribcage, and one point if white patterns were observed outside of the delimited region, to better quantify body markings (Figure 2). The modified scheme graded white from a minimum score of 0 to a maximum of 38, with a minimal effect on the original score maximum value of 36 [19]. Correlations between observer scores were evaluated using Pearson’s pairwise multivariate correlation on SAS JMP Pro V15 (SAS Institute, Cary, NC, USA).

A multiple linear regression modeling the effects of candidate loci, coat color (*MC1R* and *ASIP* genotype) and *KIT W19* on AGW was done using SAS JMP Pro V15 (SAS Institute, Cary, NC, USA). We independently evaluated the genotype impact of *MC1R*, *ASIP* and the candidate mutation with (*n* = 147 horses) and without (*n* = 135 horses) the presence of *W19* on the AGW (Table 1 and Table S2). Genotypes for all variants were obtained through the Etalon Diagnostics (Menlo Park, CA, USA) commercial testing.

## 3. Results

### 3.1. Variant Analysis Suggests a Candidate in KIT Influenced by MC1R

Exon sequencing of both stallions identified homozygosity for two non-synonymous variants, NC_009159.3:g.21551234C>G in *MITF* and NC_009146.3:g.79566881T>C in *KIT*, respectively recorded as *rs1148371483* and *rs1140732842* on the Ensembl Variation Annotation release 104 [28]. We did not observe an association between the *MITF* variant and the AGW phenotype in the 135 horses (*p* = 0.4256; *W19* individuals excluded). Functional predictions also support that the *MITF* variant, a glycine to alanine change, is not a likely candidate, as SIFT and PROVEAN predicted its effect as neutral (score = 0.23, SIFT; score = −0.356, PROVEAN) [29].

**Figure 3:**
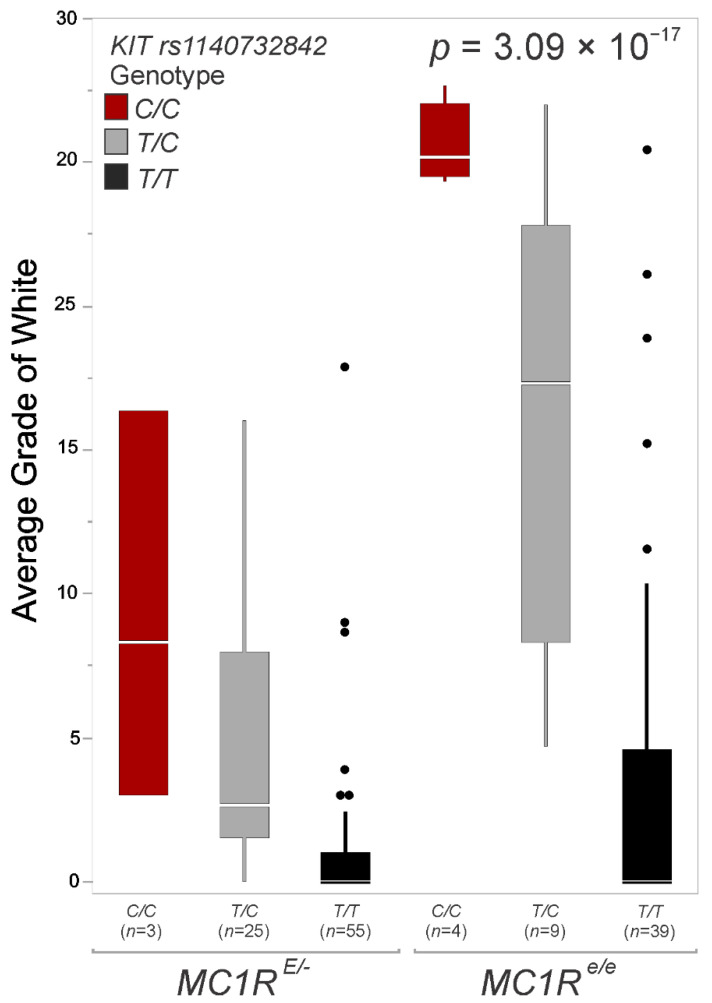
Genotype distribution of the *rs1140732842* polymorphism in 135 individuals by Average Grade of White and *MC1R* genotype (black/chestnut).

The presence of the alternate allele at *rs1140732842* is associated with a substantial effect on the AGW quantitative phenotype (F (2, 132) = 32.51, (ANOVA) *p* = 3.3 × 10^−12^) (Table 1). The *KIT* variant *rs1140732842* is predicted by the SIFT method as not tolerated (score = 0.03), and as deleterious by PROVEAN (score = −3.363). This variant substitutes an uncharged polar threonine to a nonpolar alanine in the *KIT* protein structure (p.T391A). The *MC1R* genotype alone also has a small yet significant effect on AGW (F (1, 133) = 17.99, (ANOVA) *p* = 4.12 × 10^−5^), with *e/e* (chestnut) individuals demonstrating higher AGW scores on average (mean score of 7.63) than *MC1R E*/*-* (eumelanin dominant) individuals (mean score of 2.54) (Figure 3). The *ASIP Agouti* locus has no significant effect on the AGW phenotype (Table 1). The *KIT rs1140732842* variant appears to have an incomplete penetrance autosomal dominant, or additive mode of inheritance for AGW, that could be cryptic in *MC1R E*/*-* individuals. When evaluating the effect of *W19* in AGW (Table S2, N = 147), the best-fitting model included all four loci (AICc = 919.73). Notably, the AGW scores from the three independent observers were highly correlated (*r*(147) > 0.9846, *p* < 4.38 × 10^−87^), demonstrating the repeatability of the AGW scoring methodology in this study.

### 3.2. Linkage Disequilibrium between KIT and MC1R

**Figure 4:**
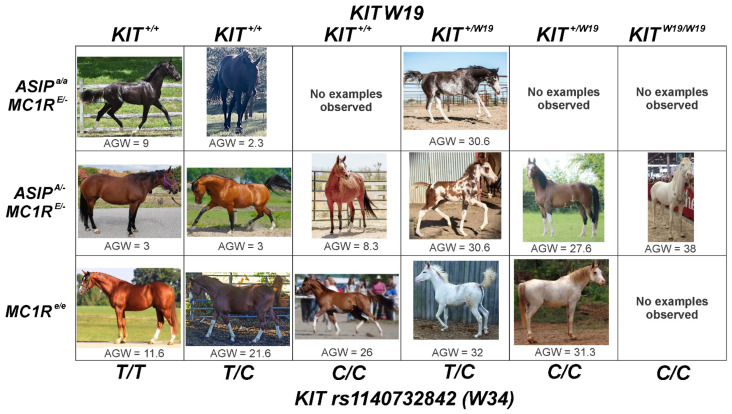
Phenotypic examples of the *rs1140732842* (*W34*) and compound *W19* allele action in different *MC1R Extension* and *ASIP Agouti* loci, as well as respective Average Grade of White (AGW) scores.

We did not observe linkage disequilibrium between *MC1R* alleles and the *KIT rs1140732842* variant in the 147 horses (*r*^2^ = 0.0001, D′ = 0.065, LOD = 0.06). Similarly, Brooks et al. [24] demonstrated that the *KIT ^W20^* allele (exon 14) was not in linkage disequilibrium with the *MC1R Extension* locus in a cohort of 364 American Paint Horses [25]. However, the *KIT W19* mutant allele (exon 8, 13.1 Kb apart) is in perfect linkage with the *rs1140732842* (exon 7) *C* variant (*r*^2^ = 0.17, D′ = 1, LOD = 6.49). Three horses demonstrated compound genotypes (EdX4400 and EdX1926: *KIT rs1140732842 C/C W19/KIT+* and EdX2927: *KIT rs1140732842 C/C W19/W19*), indicating that the *KIT W19* may have appeared after the *rs1140732842* mutant variant, as we observed heterozygosity of the *W19* allele in the presence of homozygosity of the *KIT rs1140732842 C* allele, yet not the opposite (Figure 4, File S2).

## 4. Discussion

Based on VGNC:19433 and NP_001157338.1 annotations as wild-type models, the computational analysis of the protein change in folding free energy upon mutation predicts that the p.T391A variant is destabilizing (Supplementary File S1) [30]. The amino acid threonine is highly conserved (Genomic Evolutionary Rate Profiling (GERP) score = 3.33) in this position in 91 eutherian mammals, including humans and mice [28], which could explain the observed low (1.51%) minor allele frequency of the *rs1140732842* variant in 1431 genotyped horses.

The effects of black or chestnut base coat colors on white spotting patterns were previously observed in the Arabian [31,32], the Franches-Montagnes horse [18,19] and the American Paint Horse [24]. While there is no linkage between the *MC1R Extension* locus and the candidate *KIT* variant in our cohort, the epistatic effect might be explained by other biological mechanisms. It is possible that the *MC1R e*/*e* genotype negatively affects the proliferation and differentiation of melanocytes, as observed in the murine recessive yellow (*Mc1r^e^*) model [33]. Lower activity of the *MC1R* receptor, as is likely to result from the loss-of-function variant, promotes pheomelanin production [34]. As *KIT* is also involved in melanocyte pigmentation and development, the combined effect of deleterious alleles at both loci likely promotes a higher likelihood of failed melanocyte migration or maturation, resulting in unpigmented skin devoid of melanocytes [35]. Furthermore, exon screening cannot rule out the possibility that *rs1140732842* is tagging a haplotype bearing a non-coding regulatory change in the *KIT* gene.

Individuals possessing at least one *rs1140732842* alternate allele © included the Arabian and its crosses, as well as Warmblood, Rocky Mountain Horse, American Quarter Horse, American Paint Horse, Appaloosa, Mustang, Mangalarga, Mangalarga Marchador and Morgan breeds (Table S1). Notably, the three original individuals in the *KIT W19* publication are also recorded as compound *rs1140732842* heterozygotes [5]. The *W19* allele seems to further increase the AGW (*p* = 1.88 × 10^−20^) and, given the AICc results, has some effect on the *ASIP* locus that we could not properly access in our study due to the small sample size and confounding effect of the *rs1140732842* variant. Due to the observed linkage, further evaluation of the *W19* allele’s phenotypic effects alone is suggested, along with a possible effect of base coat color, including respective genotypes at *ASIP* and *MC1R* as suggested by the model.

## 5. Conclusions

We report a white spotting QTL associated with the *KIT* variant *rs1140732842* and modified by the presence of the *MC1R* loss-of-function pheomelanin genotype in the horse, as well as the observed linkage of *KIT W19* to this variant in our population and in the original publication. We propose to designate this polymorphism as *W34*, following the standardized nomenclature for white spotting variants within the *KIT* gene. Given the *KIT rs1140732842* alternate allele’s significant association and QTL effect on white markings and its *MC1R* epistatic influence, genetic testing for this variant can be of value for horse owners that desire to select for quantitative white phenotypes.

## Figures and Tables

**Table 1:** Effect sizes of *MC1R Extension*, *ASIP Agouti* and *KIT rs1140732842* loci on AGW (*n* = 135 horses) using binomial regression.

	*AICc*	*MC1R Extension*	*ASIP Agouti*	*KIT rs1140732842*
AGW	918.99			
AGW + *MC1R Extension*	903.95	17.99		
AGW + *ASIP Agouti*	915.54		5.57	
AGW + *KIT rs1140732842*	869.13			32.51
AGW + *MC1R Extension* + *KIT rs1140732842*	843.23	30.26		40.51
AGW + *MC1R Extension* + *ASIP Agouti*	904.51	13.51	1.54	
AGW + *MC1R Extension* + *ASIP Agouti* + *KIT rs1140732842*	845.29	28.14	0.13	39.09
*p*-value (full model)		4.71 × 10^−7^	0.7199	5.09 × 10^−14^
*p*-values are given for the full model incorporating AGW and genetic effects.				

## Data Availability

Not applicable.

## Supplementary Materials

The following supporting information can be downloaded at: https://www.mdpi.com/article/10.3390/ani12151958/s1, Table S1: Genotypes and AGW phenotypic information for the 147 individuals; Table S2: Effect sizes of *MC1R Extension*, *ASIP Agouti*, *KIT W19* and *KIT rs1140732842* loci on AGW (*n* = 147 horses) using binomial regression; File S1: DUET—Protein Stability Change Upon Mutation prediction results for the *p.T391A* variant impact on protein stability; File S2: Graphical representation of the Linkage Disequilibrium heatmap between *MC1R*, *KIT W19* and *rs1140732842* (*W34*), as well as respective allele frequency and haplotype demonstration of the *W19 C* genotype (*r*^2^ = 0.17, D′ = 1, LOD = 6.49) to *W34 C* genotype in 147 horses.
