# Supplementary material for: A KIT Variant Associated with Increased White Spotting Epistatic to MC1R Genotype in Horses (Equus caballus)
Source: Animals (Basel). 2022 Aug 2;12(15):1958. doi: 10.3390/ani12151958 (PMC9367399; doi:10.3390/ani12151958)

DUET - Protein Stability Change Upon Mutation

mCSM Predicted Stability Change (ΔΔG):

-1.192 kcal/mol (*Destabilizing*)

SDM Predicted Stability Change (ΔΔG):

-0.69 kcal/mol (*Destabilizing*)

DUET Predicted Stability Change (ΔΔG):

-1.193 kcal/mol (*Destabilizing*)

Mutation:

Wild-type: **THR**
Position: **391**
Mutant-type: **ALA**
Chain: **A**
Secondary structure: **Loop or irregular**

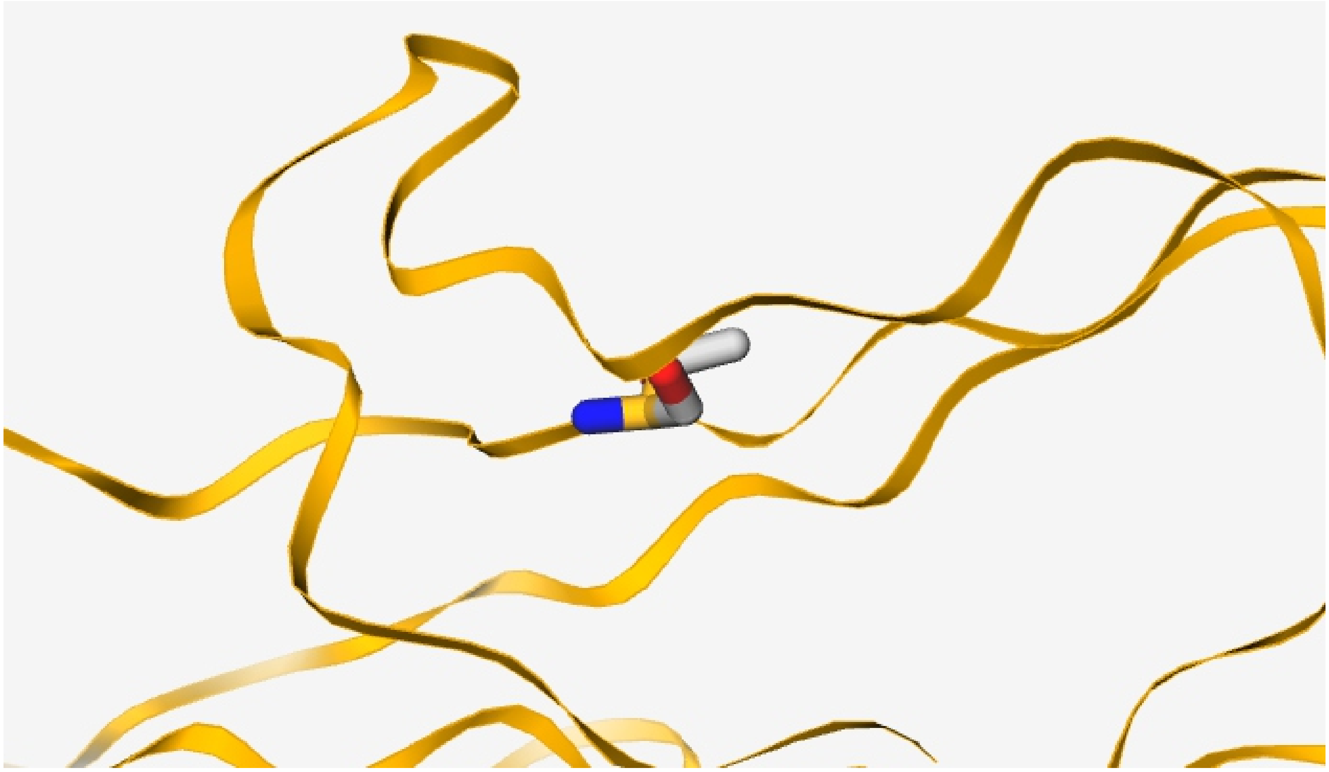

Supplement: Supplementary file 1 [file animals-12-01958-s001.zip › File S1.pdf]
