# Supplementary material for: A KIT Variant Associated with Increased White Spotting Epistatic to MC1R Genotype in Horses (Equus caballus)
Source: Animals (Basel). 2022 Aug 2;12(15):1958. doi: 10.3390/ani12151958 (PMC9367399; doi:10.3390/ani12151958)

File S2: Graphical representation of the Linkage Disequilibrium heatmap between *MC1R, KIT W19* and *rs1140732842* (*W34*), as well as respective allele frequency and haplotype demonstration of the *W19 C* genotype r^2 = 0.17, D' = 1, LOD = 6.49 to *W34 C* genotype in 147 horses.

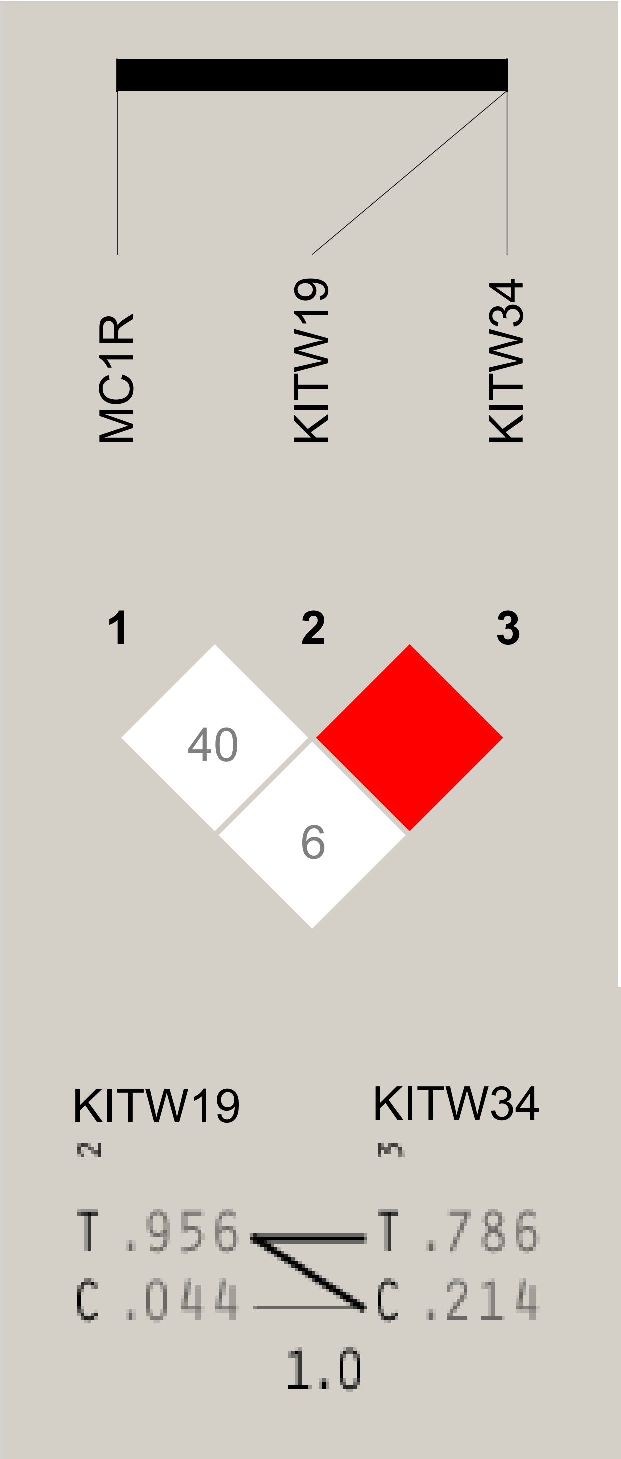

Supplement: Supplementary file 1 [file animals-12-01958-s001.zip › File S2.pdf]
