# Supplementary material for: A KIT Variant Associated with Increased White Spotting Epistatic to MC1R Genotype in Horses (Equus caballus)
Source: Animals (Basel). 2022 Aug 2;12(15):1958. doi: 10.3390/ani12151958 (PMC9367399; doi:10.3390/ani12151958)
Supplement: Supplementary file 1 [file animals-12-01958-s001.zip › Table S2.pdf]

Table S2: Effect sizes of *MC1R Extension*, *ASIP Agouti*, *KIT W19* and *KIT rs1140732842* loci on AGW (N = 147) using binomial regression.

	AICc	***MC1R Extension***	***ASIP Agouti***	***KIT W19****	***KIT rs1140732842***
AGW	1091.26				
AGW + *MC1R Extension*	1089.85	3.49			
AGW + *ASIP Agouti*	1091.13		2.20		
AGW + *KIT W19*	993.98			71.59 *	
AGW + *KIT rs1140732842*	1018.01				49.95
AGW + *MC1R Extension* + *KIT rs1140732842*	1009.46	10.78			55.67
AGW + *MC1R Extension* + *KIT W19*	977.54	19.27		86.51 *	
AGW + *MC1R Extension* + *ASIP Agouti*	1090.99	2.22	0.95		
AGW + *MC1R Extension* + *ASIP Agouti* + *KIT W19*	978.24	14.69	1.43	86.45 *	
AGW + *MC1R Extension* + *ASIP Agouti* + *KIT rs1140732842*	1010.67	11.70	0.94		55.28
AGW + 4 loci	919.73	29.03	0.26	63.93*	37.42
p-value (full model)		2.94×10^{-7}	0.6508	1.88×10^{-20}	9.56×10^{-14}

p-values are given for the full model incorporating AGW and genetic effects. * all *W19* individuals are also *KIT rs1140732842*
